# Comparison of Mediastinal Metastases of Primary Lung Cancer Versus Extrathoracic Malignancies in Patients Obtained with Endobronchial Ultrasonography-Guided Transbronchial Needle Aspiration Biopsy: A Single-Center Retrospective Study

**DOI:** 10.3390/medicina62040727

**Published:** 2026-04-10

**Authors:** Umran Ozden Sertcelik, Ebru Sengul Parlak, Habibe Hezer, Eren Goktug Ceylan, Ahmet Sertcelik, Aysegul Karalezli

**Affiliations:** 1 Department of Chest Diseases, Faculty of Medicine, Ankara Yıldırım Beyazıt University, 06800 Ankara, Türkiye; aysegulkaralezli@gmail.com; 2 Department of Chest Diseases, Ankara Bilkent City Hospital, 06800 Ankara, Türkiye; ebrusengulparlak@gmail.com (E.S.P.); hoflaz@yahoo.com (H.H.); eren807ceylan@gmail.com (E.G.C.); 3 Department of Chest Diseases, Faculty of Gülhane Medicine, Health Sciences University, 06010 Ankara, Türkiye; 4 Division of Epidemiology, Department of Infectious Diseases and Clinical Microbiology, Faculty of Medicine, Ankara Yıldırım Beyazıt University, 06800 Ankara, Türkiye; ahmetsertcelik@gmail.com; 5 Department of Infectious Diseases and Clinical Microbiology, Ankara Bilkent City Hospital, 06800 Ankara, Türkiye

**Keywords:** endobronchial sonography, fine needle aspiration, lymph node, metastasis, neoplasms

## Abstract

*Background and Objectives*: Endobronchial ultrasound-guided transbronchial needle aspiration (EBUS-TBNA) is a minimally invasive technique widely used for mediastinal staging and diagnosis in patients with lung cancer and extrathoracic malignancies. This study aimed to evaluate patient and procedural factors associated with malignant histopathological outcomes in individuals undergoing EBUS-TBNA for intrathoracic lymphadenopathy across three malignancy groups: primary lung cancer, extrathoracic solid organ malignancy, and hematological malignancy. *Materials and Methods*: This retrospective descriptive study included patients who underwent EBUS-TBNA at Ankara Bilkent City Hospital between March 2019 and December 2023. Demographic characteristics, histopathological findings, procedural details, additional sampling techniques, and imaging parameters, including FDG SUVmax values from pre-procedural PET-CT, were recorded. Histopathological outcomes were categorized as malignant or non-malignant. Binary and multinomial logistic regression analyses were performed to identify independent predictors of malignancy and to differentiate between malignancy groups and lung cancer subtypes. *Results*: A total of 776 patients underwent EBUS-TBNA, and 667 were included after excluding non-diagnostic samples. Malignancy was detected in 274 patients, including primary lung cancer (n = 213, 77.7%), extrathoracic malignancy (n = 43, 15.7%), and hematological malignancy (n = 18, 6.6%). Of the included patients, 426 (63.9%) were male; the median age was 63 (IQR = 16) years. Older age (OR = 1.03, 95% CI = 1.02–1.05, *p* < 0.001), male sex (OR = 2.05, 95% CI = 1.43–2.93, *p* < 0.001), and larger lymph node size (OR = 1.09, 95% CI = 1.06–1.11, *p* < 0.001) were independently associated with malignant outcomes. Younger age, female sex, and smaller lymph node size were associated with extrathoracic malignancy compared to primary lung cancer, while younger age was the only predictor of hematological malignancy. Larger lymph node size was inversely associated with adenocarcinoma and squamous cell carcinoma compared with small cell lung cancer. *Conclusions*: Older age, male sex, and larger lymph node size independently predict malignant EBUS-TBNA outcomes. Younger age and female sex favor extrathoracic malignancy, whereas small cell lung cancer is associated with more extensive nodal involvement. Additional bronchoscopic techniques may enhance diagnostic accuracy in selected patients.

## 1. Introduction

Intrathoracic lymphadenopathies are a common incidental finding on thoracic computed tomography and positron emission tomography in patients with primary lung malignancies or extrathoracic solid organ malignancies or hematological malignancies. The causes of mediastinal or hilar lymph node (LN) enlargement can be malignant lesions such as intrathoracic metastases of extrathoracic lesions, metastases from primary lung cancer, or involvement of the hematological malignancies; benign lesions including tuberculosis, granulomatous inflammation and reactive changes [1,2]. Histopathological confirmation of the LN for all these conditions is essential for managing patients with malignancy by staging the disease. Correctly performed mediastinal staging in non-small cell lung cancer without distant metastasis constitutes the most important step in determining the prognosis of the disease and establishing the correct treatment strategy [3,4,5,6].

Endobronchial ultrasound-guided transbronchial needle aspiration (EBUS-TBNA) is a preferred method for staging and diagnosing lung cancer. This method is not only minimally invasive but also can be performed under moderate conscious sedation [7]. In clinical practice, EBUS-TBNA is recognized as the first-line evaluation for suspicious mediastinal LNs in patients with extrathoracic malignancy. The diagnostic use of EBUS-TBNA in patients with extrathoracic solid organ malignancies with intrathoracic lymphadenopathy has increased in recent years [8,9]. However, there are few studies investigating the characteristics of EBUS-TBNA results in patients with primary lung cancer, extrathoracic solid organ malignancy, and hematological malignancy [10,11,12]. In addition to EBUS-TBNA in patients with primary lung cancer, extrathoracic solid organ malignancy, and hematological malignancy, bronchial lavage, forceps biopsy, bronchial mucosa brushing, or transthoracic fine needle aspiration biopsy may be performed during bronchoscopy to increase the diagnostic probability [13]. One of the reasons for performing additional procedures is that the use of molecularly targeted drugs and immune checkpoint inhibitors for the treatment of lung cancer in recent years has made genetic mutation research in bronchoscopic specimens increasingly important [14].

This study aimed to evaluate patient characteristics according to histopathological outcomes in patients who underwent EBUS-TBNA. Specifically, we compared patients with malignant versus non-malignant results, and among malignant cases, we further compared those with primary lung cancer, extrathoracic solid organ malignancy metastases, and hematological malignancy in terms of mediastinal LN involvement. Among patients with primary lung cancer, patient characteristics were assessed according to histopathological subtypes. In addition, the consistency of histopathological findings obtained from additional bronchoscopic and transthoracic fine needle biopsy procedures performed to increase the diagnostic probability with histopathology from EBUS-TBNA results was investigated.

## 2. Methods

### 2.1. Patients

The study was conducted as a retrospective descriptive study. Between 14 March 2019 and 31 December 2023, patients over 18 years of age who underwent EBUS performed by the Chest Diseases Clinic of Ankara Bilkent City Hospital were included in the study.

Ankara Bilkent City Hospital is a tertiary reference hospital with a total capacity of 4050 beds and 958 intensive care beds. The hospital has a Chest Diseases inpatient service with a capacity of 70 beds and 23 intensive care unit beds [15]. Approximately two to three patients are investigated in the bronchoscopy unit every weekday.

All patients over 18 years of age who underwent the EBUS-TBNA procedure with a diagnostic histopathological result in the specified date range were included in the study. Patients with missing pathological data were planned to be excluded. The histopathological results of the patients who underwent EBUS TBNA were followed up through the hospital’s electronic recording system.

### 2.2. Variables

Age, gender, forceps biopsy, bronchial lavage, bronchial brushing, and transthoracic fine needle aspiration biopsy procedures performed together with EBUS procedure and histopathological results were recorded. The stations where EBUS-TBNA was performed, ultrasonographic millimetric measurements of the LNs at the stations, the number of times TBNA was performed at the sampled station, and the total number of stations where TBNA was performed were obtained from the EBUS reports. In this retrospective study, patients were classified as malignant or non-malignant based on the histopathological results reported in the EBUS-TBNA pathology reports. Non-malignant diagnoses were established when the pathology report documented specific benign histopathological findings, including reactive lymph node hyperplasia, granulomatous inflammation, or benign lymphoid tissue, in the absence of any malignant cellular features. Since the study was retrospective in design, a standardized confirmatory follow-up protocol was not prospectively implemented for all cases classified as non-malignant.

The malignancy group was classified as primary lung cancer and extrathoracic solid organ malignancy, and hematological malignancy. For sub-analyses, primary lung cancer was grouped as small-cell lung cancer, adenocarcinoma, and squamous cell carcinoma. The total biopsied station was obtained by summing the number of stations where EBUS-TBNA was performed and the total biopsy count was obtained from the total number of samples taken. In addition to EBUS-TBNA, the number of techniques (forceps biopsy, bronchoalveolar lavage, brush biopsy, transthoracic needle biopsy) for which samples were sent for histopathology were summed, and the total additional biopsy technique was calculated. In patients undergoing EBUS-TBNA, if there was fludeoxyglucose-18 positron emission tomography-computed tomography (18F-FDG PET-CT) imaging performed in the last month before the EBUS procedure, the FDG maximum standardized uptake value (SUV-max) of the largest mediastinal LN sampled was recorded.

### 2.3. Endobronchial Ultrasonography—Transbronchial Needle Aspiration Procedure

EBUS-TBNA is performed using a convex-probe ultrasound bronchoscope, which enables real-time guidance of the transbronchial needle to mediastinal and hilar lymph nodes, as well as to parabronchial lung masses; the technical aspects encompass patient-related, technology-related, and operator-related factors. According to the CHEST Guidelines and Expert Panel Report, moderate or deep sedation is an acceptable approach during the procedure; ultrasonographic features can be used to predict malignant or benign diagnoses, but a tissue sample must still be obtained to confirm any diagnosis; and the tissue sample may be obtained with or without aspiration [16]. In this study, real-time EBUS-TBNA was performed in all enrolled patients using a convex probe EBUS bronchoscope (Model EU-ME2 7602092, Olympus Ltd., Tokyo, Japan). The procedures were conducted by three interventional pulmonologists (UOS, ESSP, HH), each possessing a minimum of two years of experience in EBUS-TBNA.

For topical anesthesia, patients first received nebulized 2% lidocaine (5 mL), followed by two puffs of 10% lidocaine spray. Additionally, a 1 mL aliquot of 2% lidocaine was instilled via the spray-as-you-go technique. Patients who opted for sedation were administered intravenous propofol and fentanyl under the supervision of an anesthesiologist. The EBUS-TBNA procedure was performed under deep sedation by anesthesiologist.

Regarding lymph node sampling, systematic EBUS-TBNA staging begins with N3 lymph node stations and progresses towards N2 and N1 stations; target lymph nodes are identified based on those with a short-axis diameter greater than 5 mm, and at least three needle passes per station are recommended; alternatively, rapid on-site cytological examination (ROSE) may be used for up to five passes until diagnostic material (malignant cells or lymphocytes) is confirmed [17]. The ROSE method, which is an optional method, was not used in our study. According to the CHEST Guidelines and Expert Panel Report, the use of a 21- or 22-gauge needle is an acceptable option in terms of needle selection [16]. In this study, LN sampling was performed using a dedicated 22-gauge TBNA needle (NA-201SX, Olympus Ltd.). The selection of LN stations and the number of aspirates per node were determined at the discretion of the operator. During the EBUS examination, all mediastinal LN stations were systematically evaluated, and the short-axis diameter was measured and documented. The interventional pulmonologist determined the appropriate target station(s) based on the preliminary diagnosis and proceeded with aspiration sampling accordingly.

EBUS-TBNA specimens were initially placed in microcassettes during the procedure and subsequently transferred to tissue cassettes for formalin fixation, paraffin embedding, hematoxylin and eosin staining, and additional immunohistochemical analyses. Notably, liquid-based cytology was not utilized for sample processing. In this study, all EBUS-TBNA procedures were performed in accordance with the technical standards outlined in this guideline. In the patients included in the study who underwent the EBUS procedure, no complications were reported during or after the procedure.

Since this study was designed as a retrospective study, histopathological results were obtained from standard pathology reports prepared by pathologists during routine clinical practice. The pathology reports were finalized independently of the interventional pulmonologists who performed the EBUS-TBNA procedures and prior to any clinical decision-making process.

### 2.4. Statistical Analysis

Categorical variables are presented as numbers and percentages. Quantitative variables were checked for normal distribution by visual (histograms, Q–Q plots) and statistical testing (Shapiro–Wilk test). Due to non-normal distribution, descriptive statistics are reported using the median (interquartile range = IQR) for quantitative variables. Pairwise comparisons were performed using Pearson’s Chi-Squared test or Fisher’s exact test for categorical variables and Mann–Whitney U test for quantitative variables. Comparisons among the primary lung cancer groups were performed using Pearson’s Chi-Squared test or Fisher–Freeman–Halton’s exact test for categorical variables and Kruskal–Wallis test for quantitative variables. Mann–Whitney U test was used as the post hoc test, and interpreted with the Bonferroni correction.

The consistency of the histopathological results obtained from EBUS-TBNA and additional techniques was evaluated and interpreted using Cohen’s Kappa test (κ).

The determinants of having a malignant histopathology finding compared to having a non-malignant histopathology finding were evaluated by binary logistic regression analysis. Model goodness of fit was tested with the Hosmer–Lemeshow test. Interaction was examined and the interaction terms were not statistically significant. The correlation between the variables was assessed to cope with the multicollinearity. A multinomial logistic regression analysis was performed to identify factors associated with the detection of mediastinal metastases from extrathoracic solid malignancies and mediastinal involvement of hematologic malignancies—using primary lung cancer as the reference category—as well as factors associated with the detection of squamous cell carcinoma and adenocarcinoma, with small cell lung cancer serving as the reference category among primary lung cancers, based on EBUS-TBNA histopathology.

A *p*-value less than 0.05 (two-sided) was considered statistically significant. Data imputation was not performed and statistical analysis was completed with valid data. Data were analyzed using Statistical Product and Service Solutions version 23.0 (IBM Corp, Armonk, NY, USA).

### 2.5. Ethical Approval

The study protocol was ethically approved by the local ethics committee (Scientific and Ethics Committee for Medical Research, date: 13 March 2024, no: TABED 1-24-77). The study was conducted in accordance with the principles of the current Declaration of Helsinki (Fortaleza, 2013). Informed consent was waived due to the retrospective study design by the ethics committee. The authors had access to information that could identify individual participants during data collection but after data collection, data was anonymized.

## 3. Results

**Figure 1:**
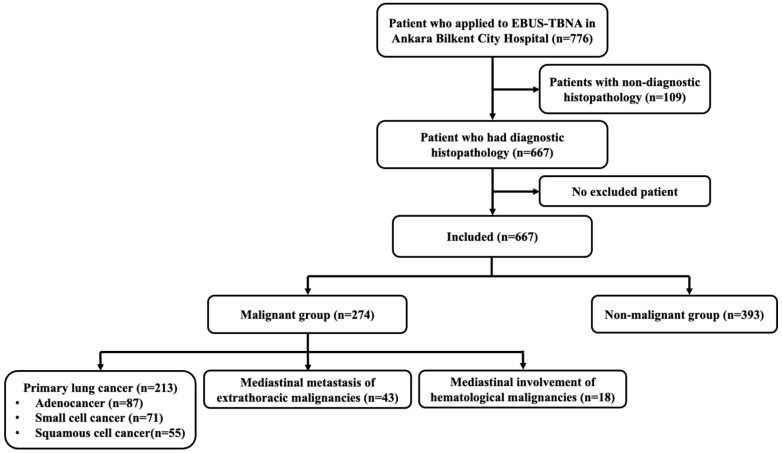
Flow chart of selection of patients who underwent to EBUS-TBNA at Ankara Bilkent City Hospital, March 2019–31 December 2023, Türkiye (n = 776).

Between 14 March 2019 and 31 December 2023, a total of 776 (89.9%) patients underwent EBUS-TBNA at Ankara Bilkent City Hospital Chest Diseases Clinic Bronchoscopy Unit. There were 109 patients with non-diagnostic histopathological results. No patient met the exclusion criteria and analyses were performed on all eligible patients (n = 667) (Figure 1).

Patients who underwent EBUS-TBNA with diagnostic histopathological results (n = 667) were grouped as malignant (n = 274) and non-malignant (n = 393). Of the included patients, 426 (63.9%) were male; the median age was 63 (IQR = 16) years. Male gender was found to be statistically higher in the malignant group (n = 203, 74.1%) compared to the non-malignant group (n = 223, 56.7%) (*p* < 0.001). The median age of the patients was higher in the malignant group (64.0, IQR = 11.0) (*p* < 0.001). Forceps biopsy (n = 84, 30.7%), brush biopsy (n = 53, 9.3%), and transthoracic needle aspiration (n = 24, 8.8%) were performed more frequently in the malignant group than in the non-malignant group (*p* < 0.001; *p* < 0.001; *p* = 0.007, respectively). When the mediastinal LN stations where EBUS-TBNA was performed were analyzed, biopsies from stations 4R (n = 92, 33.6%) and 10L (n = 75, 19.1%) were more common in the malignant group compared to the non-malignant group (*p* < 0.001; *p* = 0.046, respectively). In the non-malignant group (n = 309, 78.6%), biopsy from the subcarinal space (station 7) was statistically higher (*p* < 0.001) compared to the malignant group (n = 174, 63.5%). The size of mediastinal LNs (mm) at stations 10R, 7, and 10L, and maximum biopsied LAP size (mm) were higher in the malignant group compared to the non-malignant group (*p* = 0.030, *p* < 0.001, *p* = 0.018, *p* < 0.001, respectively). Other procedures performed in addition to EBUS-TBNA were more common in the malignant group compared to the non-malignant group (*p* < 0.001). FDG SUV-max values (12.2, IQR = 10.0) were higher in the malignant group compared to the non-malignant group (7.5, IQR = 7.2), (*p* < 0.001). EBUS-TBNA-related results for malignant and non-malignant patient groups are given in Table 1.

A total of 274 patients who underwent EBUS-TBNA were included in the study and classified into three groups: primary lung cancer (n = 213, 77.7%), extrathoracic solid organ malignancy (n = 43, 15.7%), and hematological malignancy (n = 18, 6.6%). Among the extrathoracic solid organ malignancies, breast cancer was the most common primary site (n = 15), followed by gastric, renal, and ovarian cancers, each accounting for five patients. Among the hematological malignancies, the most frequently encountered subtypes were mantle cell lymphoma (n = 8), Hodgkin’s lymphoma (n = 5), and chronic lymphocytic leukemia (CLL) (n = 3). Supplementary Table S1 shows the distribution of extrathoracic solid organ and hematological malignancies.

A total of 274 patients were included in the study: 213 (77.7%) with primary lung cancer, 43 (15.7%) with extrathoracic solid organ malignancy, and 18 (6.6%) with hematological malignancy. Male gender was predominant in the lung cancer group (80.3%) compared with the extrathoracic malignancy (44.2%) and hematological malignancy (72.2%) groups (*p* < 0.001). The lung cancer group was significantly older than the other groups (median 65.0 vs. 59.0 vs. 60.5 years, *p* = 0.001). The maximum size of the biopsied lymph node was significantly larger in the lung cancer group compared with the extrathoracic malignancy group (25.2 mm vs. 19.9 mm, *p* = 0.025). Forceps biopsy and brush biopsy were performed significantly more frequently in the lung cancer group than in the other groups (*p* < 0.001 and *p* = 0.001, respectively). The total number of additional biopsy techniques applied per patient was also significantly higher in the lung cancer group (*p* < 0.001). Other variables are presented in Table 2.

The consistency of histopathological results of EBUS-TBNA with other bronchoscopic procedures in malignant and non-malignant patients was examined and presented in Table 3. The consistency of bronchoalveolar lavage cytology with EBUS-TBNA for malignant and non-malignant patient groups was found to be fair (κ = 0.316; *p* < 0.001). The consistency of histopathological results of forceps biopsy specimens with EBUS-TBNA was substantial (κ = 0.678; *p* < 0.001) and moderate with brush biopsy (κ = 0.559; *p* < 0.001). Transthoracic needle aspiration biopsy had no statistically significant consistency with EBUS-TBNA (κ = 0.268; *p* = 0.089).

EBUS-TBNA results of the patients who underwent the EBUS-TBNA procedure and whose histopathological results were lung cancer are presented in Table 4 and Table 5. There was a statistically significant difference in forceps biopsy, brush biopsy, and transthoracic needle aspiration according to small cell lung cancer (n = 71), lung adenocarcinoma (n = 87), and squamous cell lung cancer (n = 55) groups (*p* = 0.001, *p* = 0.001, *p* = 0.019, respectively). Subcarinal (station 7) LN sampling was found to be statistically significantly different in all three groups (*p* = 0.031). The median age of the patients was older in the squamous cell carcinoma patient group than in the lung adenocarcinoma and small cell lung cancer patient groups (*p* = 0.009). In the small cell lung cancer patient group, 4R, and 7, and maximum biopsied LN size (mm) were larger than the other two groups (*p* < 0.001, *p* = 0.001, *p* < 0.001, respectively). At the 4L station, LN size was statistically smaller in the adenocarcinoma group than in the small cell cancer group (*p* = 0.040). The total number of additional procedures performed in the adenocarcinoma group was less than the other two groups (*p* < 0.001).

Binary logistic regression analysis (Model 1) revealed that older age (OR = 1.03, 95% CI = 1.02–1.05, *p* < 0.001), male gender (OR = 2.05, 95% CI = 1.43–2.93, *p* < 0.001), and larger maximum lymph node size (OR = 1.09, 95% CI = 1.06–1.11, *p* < 0.001) were independently associated with malignant histopathological outcomes. Multinomial logistic regression analysis (Model 2) showed that younger age (OR = 0.96, 95% CI = 0.92–0.99, *p* = 0.023), female gender (OR = 0.26, 95% CI = 0.12–0.52, *p* < 0.001), and smaller lymph node size (OR = 0.95, 95% CI = 0.91–0.99, *p* = 0.024) were independently associated with extrathoracic malignancy, while younger age was the only significant independent predictor of hematological malignancy (OR = 0.91, 95% CI = 0.87–0.96, *p* < 0.001), both compared with primary lung cancer. In the histological subtype comparisons (Model 3), larger lymph node size was independently associated with a lower likelihood of both adenocarcinoma (OR = 0.87, 95% CI = 0.83–0.91, *p* < 0.001) and squamous cell carcinoma (OR = 0.90, 95% CI = 0.86–0.95, *p* < 0.001) compared with small cell lung cancer. Additionally, older age was independently associated with squamous cell carcinoma compared with small cell lung cancer (OR = 1.07, 95% CI = 1.02–1.12, *p* = 0.006). Other variables are presented in Table 6.

## 4. Discussion

The first invasive diagnostic approach for mediastinal tumors was described by Daniels in 1949, who proposed the biopsy of non-palpable scalene lymph nodes as a means of diagnosing intrathoracic disorders; subsequently, cervical mediastinotomy, mediastinoscopy, and various needle biopsy techniques entered clinical practice [18]. For many years, mediastinoscopy was regarded as the gold standard for mediastinal lymph node assessment due to its well-established diagnostic accuracy, high sensitivity, and specificity; however, the requirement for general anesthesia, mandatory hospitalization, and the risk of procedure-related complications were considered significant disadvantages of this method [19]. Over the last decade, minimally invasive techniques such as EBUS-TBNA and endoscopic ultrasound-guided fine needle aspiration (EUS-FNA) have come to the forefront; owing to their higher diagnostic accuracy and lower morbidity compared with traditional surgical approaches, these techniques are now recommended as the first-line method for mediastinal staging in current clinical guidelines [20]. Therefore, in our study, we aimed to evaluate the histopathological results obtained from specimens acquired by EBUS-TBNA, a procedure that has entered more active clinical use over the last decade.

This study investigated the determinants of malignant histopathological outcomes in patients undergoing EBUS-TBNA across three distinct malignancy groups. Older age, male gender, and larger maximum lymph node size were independently associated with malignant histopathological outcomes. The independent association of male gender with malignant outcomes is consistent with the known epidemiology of lung cancer. Male gender has been independently associated with a higher prevalence of squamous cell carcinoma and poorly differentiated tumors [21]. Furthermore, larger lymph node short-axis diameter has been consistently identified as one of the most important independent predictors of malignant lymph nodes in multivariate analyses [22]. Larger lymph node size as a predictor of malignancy is also clinically consistent, as malignant infiltration is generally associated with progressive nodal enlargement.

When extrathoracic solid organ malignancy and hematological malignancy were compared with primary lung cancer, younger age and female gender emerged as independent predictors of extrathoracic solid organ malignancy. This finding is clinically plausible, as extrathoracic solid organ malignancies—most commonly breast cancer in our cohort—tend to affect younger and predominantly female patients. Females are more likely to be diagnosed at a younger age and with adenocarcinoma, whereas squamous cell and small cell lung cancers are strongly correlated with heavy smoking and are more prevalent in males [23]. Younger age was also the only significant predictor distinguishing hematological malignancy from primary lung cancer, which is consistent with the known younger age of presentation in lymphoma subtypes.

Among patients with primary lung cancer, larger lymph node size was inversely associated with both adenocarcinoma and squamous cell carcinoma when compared with small cell lung cancer. This suggests that SCLC more frequently presents with bulkier mediastinal involvement. This is consistent with the well-established clinical behavior of SCLC, which characteristically presents with bulky mediastinal lymph node involvement, and in which metastatic spread is often radiologically evident at the time of diagnosis [24]. Additionally, older age was independently associated with squamous cell carcinoma compared with small cell lung cancer, reflecting the distinct epidemiological profiles of these histological subtypes. Squamous cell carcinoma is strongly associated with smoking and older age, and tumors of this type often occur in the central part of the lung or the main airways [25].

In 2019, in a study conducted by Mehta et al. in India [26], 65 patients who underwent EBUS-TBNA procedure were included in the study, and compared to histopathology results, 20 patients (30.7%) were malignant and 45 patients (69.23%) were benign. The mean age was 62.25 years in the malignant group and 54.36 years in the benign group. FDG SUVmax values of malignant LNs proven by EBUS-TBNA were 8.9 ± 4.1, while FDG SUVmax values of benign LNs were 10.2 ± 5.57. No statistically significant difference was found between SUVmax values of malignant and non-malignant LNs [26]. In this study, the median age was found to be 65.0 years (IQR = 10.0) in the malignant patient group and 60.0 years (IQR = 20.0) in the non-malignant group. In our study, when 430 patients who underwent EBUS-TBNA and 18 F-PET-CT FDG SUV-max examinations were analyzed, there were 220 patients in the malignant group and 210 patients in the non-malignant group. FDG SUVmax was found to be 12.2 (IQR = 10.0) in the malignant group and 7.5 (IQR = 7.2) in the non-malignant group (*p* < 0.001). It is thought that Mehta et al. could not show the difference between FDG SUVmax values in the malignant and non-malignant groups due to the small number of patients in their study. Similar to the study by Mehta et al., older age was more common in the malignant patient group.

Radiotherapy plays a central role in the management of mediastinal tumors, particularly in patients with unresectable stage III non-small cell lung cancer (NSCLC). Concurrent chemoradiotherapy remains the therapeutic standard for locally advanced inoperable NSCLC, with a median overall survival in the range of 20–30 months and a five-year survival rate of approximately 30% [27]. Accurate delineation of mediastinal lymph node involvement prior to radiotherapy planning is therefore of paramount importance, and PET-CT has become an indispensable tool in this context. As involved-field irradiation of nodal hilar or mediastinal disease is the recommended standard approach rather than large mediastinal volume irradiation, PET-CT is critical to encompassing involved areas [28]. In the landmark PET-Plan phase III trial, radiotherapy planning based on 18F-FDG PET-CT with omission of PET-negative lymph nodes resulted in a more than two-fold reduction in locoregional progression at one year, and this approach may therefore be considered a current standard of care; however, PET-CT-negative but enlarged lymph nodes should always be subjected to pathological staging with EBUS depending on their location [29].

In a study conducted in 2014 in Türkiye, FDG SUVmax values of 52 mediastinal LNs were compared and SUVmax values of 35 malignant LNs were 10.23 ± 6.23 (min–max = 3.1–29) and SUVmax values of 18 benign LNs were 5.42 ± 3.87 (min–max = 2.9–18.5) (*p* < 0.05). In the same study, when the mean short diameter of LNs measured by EBUS was analyzed, it was found that the size of malignant LNs was 24.1 ± 10.7 mm (min–max = 11–46), which was 12.1 ± 4.0 mm (min–max = 6–20) larger than the size of benign LNs (*p* < 0.05) [30]. It is generally known and accepted that LNs with malignant features have higher SUVmax values than benign pathologies. In parallel with this knowledge and studies in the literature, in our study, higher FDG SUVmax values and larger size of LNs were found in the malignant group compared to the non-malignant group.

In a study of 80 patients who underwent EBUS-TBNA in China in 2022, extrathoracic malignancy with intrathoracic LN metastasis was found in 50 (62.5%) patients, primary lung cancer with nodal involvement in 14 (17.5%) patients, and benign pathologies such as tuberculosis, sarcoidosis, and reactive lymphadenitis in 16 (20.0%) patients. The characteristics associated with the malignant status of mediastinal LNs were analyzed and according to the results of multivariate analysis, it was found to be associated with the sampled LN size (longer short axis of the LN) (OR = 1.200; 95% CI = 1.024–1.407; *p* = 0.024) [8]. A study published in 2012 included a total of 48 cases, all diagnosed with extrathoracic malignancy, with a mean age of 57.4 ± 11.6 years. The mean short-axis diameter of the aspirated LNs was reported as 1.51 ± 0.63 cm. Histopathological analysis of EBUS-TBNA samples revealed malignancy in 15 cases (31.2%), tuberculosis in six cases (12.5%), sarcoidosis in four cases (8.3%), and reactive adenitis in 23 cases (48%) [31]. In this study, similar to previous studies, it was found that the sampled mediastinal LN size was larger in both the malignant patient group (median = 24.7, IQR = 13.2; *p* < 0.001) and in the subgroup of patients with primary lung cancer (median = 25.2, IQR = 13.2; *p* = 0.018) compared to the intrathoracic LN metastases of non-malignant patients and patients with extrathoracic malignancy. In addition, according to the results of binary logistic regression analysis, an increase of 1 mm in the LN in the malignant patient group compared to the non-malignant group increased the malignancy status 1.09 times (OR = 1.09, 95% CI = 1.06–1.11, *p* < 0.001); similarly, in patients diagnosed with primary lung cancer compared to extrathoracic malignancies with mediastinal LN metastasis, an increase of 1 mm in the LN increased the status of primary lung cancer by 1.04 times (OR = 1.04, 95% CI = 1.00–1.08, *p* = 0.049).

In a study by Tertemiz et al., which analyzed the results of EBUS-TBNA performed in patients with extrathoracic malignancies in whom 91 mediastinal LNs were detected, no malignancy was observed in 54 patients (59.3%), metastasis of extrathoracic malignancy was found in 33 patients (36.3%) and metastasis of primary lung cancer was found in four patients (4.4%). Among extrathoracic malignancies with mediastinal LNs showing malignant features, laryngeal carcinoma was found in nine patients, breast cancer in five patients, and colon cancer in seven patients [11]. In this study, among the extrathoracic malignancies with mediastinal LN metastasis, breast cancer was found in 15 patients, mantle cell lymphoma in eight patients, gastric cancer, Hodgkin lymphoma, renal cell cancer, and ovarian cancer in five patients each. Similar to the literature, mediastinal LN metastasis was more common in breast cancer. It is thought that the presence of different diagnoses may be due to the variability of the patient profile followed by the centers where the procedure is performed.

In this study, non-malignant diagnoses were based exclusively on EBUS-TBNA pathology reports documenting benign histopathological findings such as reactive lymph node hyperplasia, granulomatous inflammation, or benign lymphoid tissue. It is well recognized that EBUS-TBNA carries an inherent risk of false-negative results, particularly in cases where adequate tissue sampling may not have been achieved or where malignant cells are sparsely distributed within the lymph node. In the absence of a systematic confirmatory follow-up protocol—including repeat biopsy, surgical pathology, or radiological stability assessment over time—a subset of cases classified as non-malignant in this study may potentially represent false-negative EBUS-TBNA results. This possibility should be taken into account when interpreting the diagnostic performance data and the results of the regression analyses presented in this study. Nevertheless, this reflects the inherent nature of retrospective study designs and is a recognized challenge in studies evaluating the diagnostic yield of minimally invasive sampling techniques.

In this study, when the consistency of histopathological results of EBUS-TBNA procedure with forceps biopsy, brush biopsy, bronchoalveolar lavage, and transthoracic fine needle aspiration biopsy was examined, it was found to be substantial consistency for forceps biopsy (κ = 0.678; *p* < 0.001) and moderate consistency for brush biopsy (κ = 0.559; *p* < 0.001). It is thought that these additional interventions may increase the probability of diagnosis in cases with difficult diagnoses. Although it is believed that the low number of patients who underwent simultaneous transthoracic fine needle aspiration may be effective in the lack of a statistically significant consistency, it is also thought that the fact that the sampling targets of transthoracic fine needle aspiration biopsy and EBUS-TBNA procedures are in a different location may also be effective [13,32]. Although it is not exactly comparable, 94 patients with malignant diagnosis were included in the study published in 2023 in which the diagnostic yield of transbronchial lung cryobiopsy (TBLC) between forceps biopsy cytology and brushing cytology in Japan was examined. While the diagnostic yield for TBLC, forceps biopsy, and brushing cytology was 86/81/82%, the diagnostic yield was 94% when all procedures were combined [33].

### 4.1. Strengths

This study, which investigated the factors associated with LNs with malignant histopathological results in EBUS-TBNA samples, has a larger sample size than similar studies. The ability to collect data in a short period with this large sample size makes this study stand out in terms of limiting the confounding effects of time. Moreover, as far as we know, there is no one-to-one comparable study in the literature. Although this study was descriptive, it strengthened its findings by controlling for confounding factors. Several additional features further distinguish this study from previously published work. First, unlike most prior studies that focused on a single patient group, the present study simultaneously evaluated three distinct groups: patients with primary lung cancer, extrathoracic solid organ malignancies, and hematological malignancies. This design allowed a direct diagnostic comparison between these clinically distinct groups within the same cohort. Second, PET-CT-derived variables, including FDG SUVmax values from pre-procedural imaging and lymph node short-axis diameters measured during EBUS, were analyzed in relation to histopathological outcomes. This multimodal approach has rarely been used in studies focusing on this patient population. Third, this study not only evaluated the diagnostic yield of EBUS-TBNA in isolation, but also assessed the consistency of histopathological findings obtained from additional bronchoscopic procedures—including bronchial lavage, forceps biopsy, bronchial mucosa brushing, and transthoracic fine needle aspiration biopsy—with those obtained from EBUS-TBNA. To our knowledge, no previous study has simultaneously addressed all of these aspects within a single cohort.

### 4.2. Limitation

Limitations of our study include its retrospective single-center design with limited sample size. While one of the most important problems in retrospective studies is the high rate of missing data, the missing data in this study is quite low. Due to being a single-center study, this limits the generalizability of the results. Second, since this study was retrospective, a formal pathologist blinding procedure was not implemented. Histopathological results were obtained from routine clinical pathology reports, which were finalized independently of the procedural team; however, the absence of prospective blinding remains a methodological limitation. Third, rapid on-site evaluation (ROSE) was not available at our institution during the study period and was therefore not used in any of the procedures. The absence of ROSE may have affected sample adequacy assessment and potentially influenced the number of passes performed per lymph node. Fourth, the classification of non-malignant cases was based solely on EBUS-TBNA pathology reports, without a standardized confirmatory follow-up protocol such as repeat biopsy, surgical pathology, or radiological stability assessment over time. Therefore, the possibility that a subset of non-malignant cases may represent false-negative EBUS-TBNA results cannot be entirely excluded, which may introduce verification bias into the regression analyses. Finally, while this study provides estimates based on patient characteristics, the experience of the interventional pulmonologist performing the procedure and the pathologist performing the histopathological examination should also be considered.

### 4.3. Future Directions

The optimal management of negative or non-diagnostic EBUS-TBNA findings in patients with suspected extrathoracic malignancy has not yet been clearly defined, and prospective studies with standardized follow-up protocols are needed. The role of PET-CT SUVmax values in guiding lymph node selection should be further evaluated, as defining reliable thresholds may improve diagnostic efficiency. Finally, tissue acquisition protocols need to evolve to meet the growing demands of molecular testing in modern oncology. The integration of complementary techniques, such as intranodal forceps biopsy, into routine practice may help address this challenge and should be assessed in future studies.

## 5. Conclusions

EBUS-TBNA remains the first-line minimally invasive method for mediastinal lymph node evaluation in patients with both primary lung cancer and extrathoracic malignancies. This study demonstrated that older age, male gender, and larger lymph node size are independent predictors of malignant histopathological outcomes. Younger age and female gender favor extrathoracic solid organ malignancy over primary lung cancer, while younger age also distinguishes hematological malignancy from primary lung cancer. Among lung cancer histological subtypes, small cell lung cancer is associated with bulkier mediastinal lymph node involvement compared with adenocarcinoma and squamous cell carcinoma. These findings may assist clinicians in predicting the likelihood of malignancy and differentiating between malignancy types prior to histopathological confirmation. Forceps and brush biopsy showed substantial and moderate agreement with EBUS-TBNA results, respectively, and may serve as useful complementary procedures to increase diagnostic yield in selected cases. Overall, a multimodal bronchoscopic approach combining EBUS-TBNA with additional biopsy techniques, guided by clinical and radiological findings, may optimize diagnostic accuracy in patients with intrathoracic lymphadenopathy.

## Figures and Tables

**Table 1:** Distribution of malignant and non-malignant patient characteristics based on endobronchial ultrasonography-guided transbronchial needle aspiration results.

	Total (n = 667)	Malignant (n = 274)	Non-Malignant (n = 393)		
	n (%)	n (%)	n (%)	OR (95% CI)	*p*-Value
Male gender	426 (63.9)	203 (74.1)	223 (56.7)	2.18 (1.56–3.05)	<0.001
Bronchoalveolar lavage	544 (81.6)	227 (82.8)	317 (80.7)	1.16 (0.78–1.73)	0.474
Forceps biopsy	131 (19.6)	84 (30.7)	47 (12.0)	3.26 (2.19–4.85)	<0.001
Brush biopsy	72 (10.8)	53 (19.3)	19 (4.8)	4.72 (2.72–8.18)	<0.001
Transthoracic needle aspiration	39 (5.8)	24 (8.8)	15 (3.8)	2.42 (1.25–4.70)	0.007
EBUS-TBNA					
4R	167 (25.0)	92 (33.6)	75 (19.1)	2.14 (1.50–3.06)	<0.001
10R	70 (10.5)	36 (13.1)	34 (8.7)	1.60 (0.97–2.62)	0.060
11R	78 (11.7)	26 (9.5)	52 (13.2)	0.69 (0.42–1.13)	0.139
7	483 (72.4)	174 (63.5)	309 (78.6)	0.47 (0.34–0.69)	<0.001
4L	23 (3.4)	14 (5.1)	9 (2.3)	2.30 (0.98–5.39)	0.050
10L	71 (10.6)	37 (13.5)	34 (8.7)	1.65 (1.01–2.70)	0.046
11L	60 (9.0)	21 (7.7)	39 (9.9)	0.75 (0.43–1.31)	0.316
	**Malignant**		**Non-malignant**		
	**n**	**Median (IQR)**	**n**	**Median (IQR)**	***p*-Value**
Age (years)	274	64.0 (11.0)	393	60.0 (20.0)	<0.001
4R, mm	92	19.3 (14.1)	75	12.8 (9.1)	<0.001
4R, biopsy count	92	5.0 (2.0)	75	5.0 (2.0)	0.770
10R, mm	36	24.5 (16.9)	34	17.2 (9.1)	0.030
10R, biopsy count	36	6.0 (2.0)	34	5.0 (1.3)	0.493
11R, mm	26	18.0 (17.0)	52	14.8 (8.9)	0.081
11R, biopsy count	26	5.5 (3.0)	52	5.5 (2.4)	0.863
7, mm	174	22.6 (14.2)	309	17.7 (12.5)	<0.001
7, biopsy count	174	6.0 (2.0)	308	6.0 (2.0)	0.316
4L, mm	14	15.3 (14.3)	9	13.6 (13.9)	0.369
4L, biopsy count	14	5.0 (2.0)	9	5.0 (3.0)	0.926
10L, mm	37	20.1 (12.3)	34	14.8 (12.5)	0.018
10L, biopsy count	37	6.0 (3.0)	34	5.0 (2.0)	0.126
11L, mm	21	12.7 (14.6)	39	11.9 (5.6)	0.333
11L, biopsy count	21	6.0 (2.0)	39	6.0 (2.0)	0.459
Total biopsy count	274	8.0 (4.0)	392	7.0 (4.0)	0.069
Maximum biopsied lymph node, mm	274	24.7 (13.2)	393	17.7 (12.1)	<0.001
Total biopsied station with EBUS-TBNA	274	1.0 (1.0)	393	1.0 (1.0)	0.245
Total additional biopsy technique	274	1.0 (1.0)	393	1.0 (0.0)	<0.001
18 F-PET-CT FDG SUV-max	220	12.2 (10.0)	210	7.5 (7.2)	<0.001

IQR: interquartile range, EBUS-TBNA: Endobronchial ultrasound-transbronchial needle aspiration, 18 F-PET-CT FDG SUV-max: fludeoxyglucose-18 positron emission tomography-computed tomography maximum standardized uptake value.

**Table 2:** Distribution of primary lung cancer, extrathoracic solid organ malignancy, and hematological malignancy patient characteristics based on endobronchial ultrasonography-guided transbronchial needle aspiration results.

	Total (n = 274)		Primary Lung Cancer (n = 213)		Extrathoracic Solid Organ Malignancies (n = 43)		Hematological Malignancies (n = 18)		
	n (%)		n (%)		n (%)		n (%)		*p*-Value
Male gender	203 (74.1)		171 ^a^ (80.3)		19 ^b^ (44.2)		13 ^a,b^ (72.2)		<0.001
Bronchoalveolar lavage	227 (82.8)		185 ^a^ (86.9)		29 ^b^ (67.4)		13 ^a,b^ (72.2)		0.004
Forceps biopsy	84 (30.7)		81 ^a^ (38.0)		3 ^b^ (7.0)		- ^b^		<0.001
Brush biopsy	53 (19.3)		51 ^a^ (23.9)		2 ^b^ (4.7)		- ^a,b^		0.001
Transthoracic needle aspiration	24 (8.8)		22 (10.3)		2 (4.7)		-		0.269 *
EBUS-TBNA									
4R	92 (33.6)		70 (32.9)		12 (27.9)		10 (55.6)		0.102
10R	36 (13.1)		31 (14.6)		3 (7.0)		2 (11.1)		0.393
11R	26 (9.5)		22 (10.3)		3 (7.0)		1 (5.6)		0.864 *
7	174 (63.5)		134 (62.9)		30 (69.8)		10 (55.6)		0.535
4L	14 (5.1)		13 (6.1)		-		1 (5.6)		0.199 *
10L	37 (13.5)		29 (13.6)		6 (14.0)		2 (11.1)		0.952
11L	21 (7.7)		17 (8.0)		2 (4.7)		2 (11.1)		0.697 *
	**n**	**Median (IQR)**	**n**	**Median (IQR)**	**n**	**Median (IQR)**	**n**	**Median (IQR)**	***p*-Value**
Age (years)	274	64.0 (11.0)	213	65.0 ^a^ (10.0)	43	59.0 ^b^ (16.0)	18	60.5 ^b^ (18.5)	0.001
4R, mm	92	19.3 (14.1)	70	18.7 (15.0)	12	20.4 (13.6)	10	25.8 (14.2)	0.619
4R, biopsy count	92	5.0 (2.0)	70	5.0 (2.0)	12	6.0 (1.8)	10	5.0 (2.8)	0.308
10R, mm	36	24.5 (16.9)	31	25.7 (16.9)	3	14.3 (-)	2	15.9 (-)	0.499
10R, biopsy count	36	6.0 (2.0)	31	6.0 (2.0)	3	5.0 (-)	2	4.0 (-)	0.580
11R, mm	26	18.0 (17.0)	22	18.6 (17.0)	3	16.7 (-)	1	-	0.886
11R, biopsy count	26	5.5 (3.0)	22	5.5 (3.0)	3	6.0 (-)	1	-	0.848
7, mm	174	22.6 (14.2)	134	23.0 (14.6)	30	20.8 (12.3)	10	23.9 (13.9)	0.466
7, biopsy count	174	6.0 (2.0)	134	6.0 (2.0)	30	6.0 (2.0)	10	6.0 (1.5)	0.702
4L, mm	14	15.3 (14.3)	13	15.0 (14.0)	-	-	1	-	0.106
4L, biopsy count	14	5.0 (2.0)	13	5.0 (2.0)	-	-	1	-	0.896
10L, mm	37	20.0 (12.3)	29	20.9 (12.3)	6	14.2 (8.8)	2	19.9 (-)	0.070
10L, biopsy count	37	6.0 (3.0)	29	6.0 (2.0)	6	4.0 (3.3)	2	4.5 (-)	0.062
11L, mm	21	12.7 (14.6)	17	11.1 (13.3)	2	23.2 (-)	2	20.1 (-)	0.120
11L, biopsy count	21	6.0 (2.0)	17	6.0 (2.5)	2	6.0 (-)	2	6.5 (-)	0.647
Total biopsy count	274	8.0 (4.0)	213	8.0 (4.0)	43	7.0 (4.0)	18	8.5 (5.0)	0.385
Maximum size of biopsied lymph node, mm	274	24.1 (13.2)	213	25.2 ^a^ (13.2)	43	19.9 ^b^ (12.0)	18	23.9 ^a,b^ (11.5)	0.025
Total biopsied station with EBUS-TBNA	274	1.0 (1.0)	213	1.0 (1.0)	43	1.0 (1.0)	18	2.0 (1.0)	0.347
Total additional biopsy technique	274	1.0 (1.0)	213	1.0 ^a^ (1.0)	43	1.0 ^b^ (1.0)	18	1.0 ^b^ (1.0)	<0.001
18 F-PET-CT FDG SUV-max	220	12.1 (10.0)	170	12.3 (9.3)	35	11.2 (12.6)	15	11.0 (11.1)	0.800

OR: odds ratio, CI: confidence interval, IQR: interquartile range, EBUS-TBNA: Endobronchial ultrasound-transbronchial needle aspiration, 18 F-PET-CT FDG SUV-max: fludeoxyglucose-18 positron emission tomography-computed tomography maximum standardized uptake value. Statistically significant differences between groups are shown with lowercase superscripts. * Fisher–Freeman–Halton’s exact test.

**Table 3:** The consistency of malignancy status of patients undergoing EBUS-TBNA between additional diagnostic procedures.

		EBUS-TBNA	
		Malignant n (%)	Non-Malignant n (%)
Bronchoalveolar lavage cytology	Malignant	66 (29.1)	2 (0.6)
	Non-malignant	161 (70.9)	315 (99.4)
κ	0.316	*p*-value	<0.001
Forceps bx	Malignant	68 (81.9)	5 (10.9)
	Non-malignant	15 (18.1)	45 (89.1)
κ	0.678	*p*-value	<0.001
Brush bx	Malignant	42 (79.2)	3 (15.8)
	Non-malignant	11 (20.8)	16 (84.2)
κ	0.559	*p*-value	<0.001
Transthoracic needle aspiration bx	Malignant	19 (79.2)	8 (50.0)
	Non-malignant	5 (20.8)	8 (50.0)
κ	0.268	*p*-value	0.089

**Table 4:** Patient characteristics according to subtypes of patients with lung cancer.

	Total (n = 213)	Adenocarcinoma (n = 87)	Small Cell Cancer (n = 71)	Squamous Cell Cancer (n = 55)	
	n (%)	n (%)	n (%)	n (%)	*p*-Value
Male gender	171 (80.3)	67 (77.0)	59 (83.1)	45 (81.8)	0.599
BAL	185 (86.9)	73 (83.9)	64 (90.1)	48 (87.3)	0.511
Forceps biopsy	51 (23.9)	10 ^a^ (11.5)	21 ^b^ (29.6)	20 ^b^ (36.4)	0.001
Brush biopsy	51 (23.9)	10 ^a^ (11.5)	21 ^b^ (29.6)	20 ^b^ (36.4)	0.001
Transthoracic needle asp	22 (10.3)	15 ^a^ (17.2)	3 ^b^ (4.2)	4 ^a,b^ (7.3)	0.019
EBUS-TBNA					
4R	70 (32.9)	29 (33.3)	23 (32.4)	18 (32.7)	0.992
10R	31 (14.6)	9 (10.3)	12 (16.9)	10 (18.2)	0.344
11R	22 (9.1)	8 (9.2)	8 (11.3)	6 (10.9)	0.901
7	134 (62.9)	63 ^a^ (72.4)	43 ^a,b^ (60.6)	28 ^b^ (50.9)	0.031
4L	13 (6.1)	4 (4.6)	4 (5.6)	5 (9.1)	0.510 *
10L	29 (13.6)	9 (10.3)	11 (15.5)	9 (16.4)	0.507
11L	17 (8.0)	9 (10.3)	7 (9.9)	1 (1.8)	0.146

EBUS-TBNA: Endobronchial ultrasound-transbronchial needle aspiration, BAL: Bronchoalveolar lavage, asp.: aspiration. Statistically significant differences between groups are shown with lowercase superscripts. * Fisher–Freeman–Halton’s exact test.

**Table 5:** Patient characteristics according to subtypes of patients with lung cancer.

	Adenocarcinoma (n = 87)		Small Cell Cancer (n = 71)		Squamous Cell Cancer (n = 55)		
	n	Median (IQR)	n	Median (IQR)	n	Median (IQR)	*p*-Value
Age (years)	87	65.0 (11.0) ^a^	71	65.0 (9.0) ^a^	55	68.0 (12.0) ^b^	0.009
4R, mm	29	16.1 (9.2) ^a^	23	28.6(13.9) ^b^	18	17.0 (8.0) ^a^	<0.001
4R, biopsy count	29	5.0 (3.0)	23	5.0 (2.0)	18	5.0 (2.3)	0.766
10R, mm	9	20.5 (16.6)	12	28.6 (8.6)	10	22.8 (19.1)	0.263
10R, biopsy count	9	6.0 (2.5)	12	6.0 (1.5)	10	5.5 (2.3)	0.899
11R, mm	8	17.5 (13.4)	8	23.5 (18.5)	6	18.7 (18.5)	0.408
11R, biopsy count	8	5.0 (4.3)	8	6.0 (2.5)	6	4.5 (3.5)	0.316
7, mm	63	21.8 (14.5) ^a^	43	28.3 (15.0) ^b^	28	22.4 (14.5) ^a^	0.001
7, biopsy count	63	6.0 (2.0)	43	6.0 (2.0)	28	6.0 (1.8)	0.261
4L, mm	4	14.0 (4.8) ^a^	4	27.7 (13.4) ^b^	5	14.5 (10.8) ^a,b^	0.040
4L, biopsy count	4	5.0 (2.3)	4	5.0 (1.5)	5	4.0 (2.5)	0.884
10L, mm	9	18.2 (4.9)	11	22.0 (14.0)	9	27.0 (17.4)	0.404
10L, biopsy count	9	5.0 (4.0)	11	6.0 (3.0)	9	6.0 (2.0)	0.840
11L, mm	9	10.0 (8.2)	7	11.8 (25.4)	1	-	0.159
11L, biopsy count	9	6.0 (2.0)	7	6.0 (3.0)	1	-	0.290
Total biopsy count	87	8.0 (3.0)	71	8.0 (5.0)	55	7.0 (3.0)	0.076
Maximum size of biopsied lymph node, mm	87	21.1 (12.9) ^a^	71	31.0 (10.0) ^b^	55	23.3 (12.2) ^a^	<0.001
Total biopsied station with EBUS-TBNA	87	1.0 (1.0)	71	1.0 (1.0)	55	1.0 (1.0)	0.417
Total additional biopsy technique	87	1.0 (1.0) ^a^	71	2.0 (2.0) ^b^	55	2.0 (2.0) ^b^	<0.001
18 F-PET-CT FDG SUV-max	71	11.5 (9.5)	52	12.7 (6.7)	47	14.6 (13.1)	0.587

IQR: interquartile range, EBUS-TBNA: Endobronchial ultrasound-transbronchial needle aspiration, 18 F-PET-CT FDG SUV-max: fludeoxyglucose-18 positron emission tomography-computed tomography maximum standardized uptake value. Statistically significant differences between groups are shown with lowercase superscripts.

**Table 6:** Determinants of malignant histopathological outcomes versus non-malignant pathologies (Model 1), extrathoracic solid organ malignancy and hematological malignancy versus primary lung cancer (Model 2), and histological subtype comparisons within primary lung cancer (Model 3), assessed by binary and multinomial logistic regression analyses.

	Model 1			Model 2						Model 3					
	Malignant vs. Non-Malignant			Extrathoracic vs. Primary			Hematological vs. Primary			Adenocarcinoma vs. SCLC			SCC vs. SCLC		
	OR	95% CI	*p*-Value	OR	95% CI	*p*-Value	OR	95% CI	*p*-Value	OR	95% CI	*p*-Value	OR	95% CI	*p*-Value
Age, years	1.03	1.02–1.05	<0.001	0.96	0.92–0.99	0.023	0.91	0.87–0.96	<0.001	1.00	0.96–1.04	0.853	1.07	1.02–1.12	0.006
Male gender	2.05	1.43–2.93	<0.001	0.26	0.12–0.52	<0.001	1.06	0.33–3.44	0.947	0.67	0.28–1.62	0.374	0.78	0.29–2.08	0.616
Maximum size of biopsied lymph node, mm	1.09	1.06–1.11	<0.001	0.95	0.91–0.99	0.024	0.99	0.93–1.06	0.774	0.87	0.83–0.91	<0.001	0.90	0.86–0.95	<0.001

OR: odds ratio, CI: confidence interval, SCLC: small cell lung cancer, SCC: squamous cell cancer.

## Data Availability

The datasets used and/or analyzed during the current study are available from the corresponding author on reasonable request.

## Supplementary Materials

The following supporting information can be downloaded at https://www.mdpi.com/article/10.3390/medicina62040727/s1, Table S1: Distribution of extrathoracic solid organ malignancies with mediastinal lymph node metastasis, and mediastinal involvement of hematological malignancies.
