# Supplementary material for: Comparison of Mediastinal Metastases of Primary Lung Cancer Versus Extrathoracic Malignancies in Patients Obtained with Endobronchial Ultrasonography-Guided Transbronchial Needle Aspiration Biopsy: A Single-Center Retrospective Study
Source: Medicina (Kaunas). 2026 Apr 10;62(4):727. doi: 10.3390/medicina62040727 (PMC13117326; doi:10.3390/medicina62040727)
Supplement: Supplementary file 1 [file medicina-62-00727-s001.zip › medicina-4233167-supplementary.pdf]

medicina

MDPI

Supplement Table S1. Distribution of extrathoracic solid organ malignancies with mediastinal lymph node metastasis, and mediastinal involvement of hematological malignancies.

**Extrathoracic malignant**	**n=61**
Breast cancer	15
Mantle cell lymphoma	8
Gastric cancer	5
Hodgkin's lymphoma	5
Ovary cancer	5
Renal cell cancer	5
Gastrointestinal stromal tumor	3
Chronic lymphocytic leukemia	3
Thyroid papillary cancer	3
Colon cancer	2
Prostate cancer	2
Pancreas cancer	2
Giant cell lymphoma	1
Ureter cancer	1
B cell lymphoma	1
